# SOX2 activation predicts prognosis in patients with head and neck squamous cell carcinoma

**DOI:** 10.1038/s41598-018-20086-w

**Published:** 2018-01-26

**Authors:** Ji Hyun Chung, Hae Rim Jung, Ah Ra Jung, Young Chan Lee, Moonkyoo Kong, Ju-Seog Lee, Young-Gyu Eun

**Affiliations:** 1 Department of Otolaryngology-Head and Neck Surgery, School of Medicine, Kyung Hee University, Seoul, South Korea; 2 Department of Radiation Oncology, School of Medicine, Kyung Hee University, Seoul, South Korea; 3 Department of Systems Biology, The University of Texas MD Anderson Cancer Center, Houston, Texas, USA

## Abstract

SOX2 copy number and mRNA expression were analysed to examine the clinical significance of SOX2 activation in HNSCC. Gene expression signatures reflecting SOX2 activation were identified in an HNSCC cohort. Patients with HNSCC were classified into two subgroups according to the gene expression signature: SOX2-high and SOX2-low. The clinical significance of SOX2 activation was further validated in two independent cohorts. Moreover, clinical significance of SOX2 activation in response to radiotherapy was assessed in patients with HNSCC. The relationship between SOX2 activation and radiotherapy was validated in an *in vitro* experiment. Patients in the SOX2-high subgroup had a better prognosis than patients in the SOX2-low subgroup in all three patient cohorts. Results of multivariate regression analysis showed that SOX2 signature was an independent predictor of the overall survival of patients with HNSCC (hazard ratio, 1.45; 95% confidence interval, 1.09–1.92; P = 0.01). Interestingly, SOX2 activation was a predictor of therapy outcomes in patients receiving radiotherapy. Moreover, SOX2 overexpression enhanced the effect of radiotherapy in HNSCC cell lines. SOX2 activation is associated with improved prognosis of patients with HNSCC and might be used to predict which patients might benefit from radiotherapy.

## Introduction

Head and neck squamous cell carcinoma (HNSCC) most commonly arises from the mucosa of the oral cavity, pharynx, and larynx, and is the sixth most common cancer worldwide, with an incidence rate of approximately 600,000 patients per year^1^. Despite advances in our knowledge of the epidemiology and pathogenesis of HNSCC and its treatment modalities, survival rates of patients with HNSCC have not improved over the past four decades, with the 5-year survival rate of patients with HNSCC remaining at 50%^2,3^. Moreover, detailed understanding of the biology of HNSCC is needed because of the considerable clinicopathological heterogeneity among tumours.

Sex determining region Y-box 2 gene (SOX2), located at chromosome 3q26.33, encodes a transcription factor containing a high mobility group DNA-binding domain^4^. Functionally, SOX2 maintains stem cell pluripotency and determines cell fate. Moreover, ectopic expression and amplification of SOX2 are associated with cancer development^5^.

Several studies have indicated that SOX2 is associated with the development of various malignant tumours, including glioblastoma, small-cell lung cancer, and different types of squamous cell carcinomas (SCCs)^6–9^. SOX2 expression is also reported in HNSCC. However, the role of SOX2 in HNSCC remains unclear. Some studies indicate that SOX2 expression promotes the invasiveness of HNSCC cells and that high SOX2 protein levels are closely associated with poor prognosis of patients with HNSCC^10–12^. However, Züllig *et al*. reported that low SOX2 expression is significantly associated with the poor survival of patients with HNSCC^13^. Therefore, additional studies are required to validate the role of SOX2 in HNSCC and to determine treatment approaches for HNSCC patients.

In the present study, we systematically characterised genomic data of patients with HNSCC to determine the molecular subtypes associated with SOX2 activation and prognosis of patients with HNSCC. Moreover, we investigated the clinical relevance of SOX2 activation in response to radiotherapy (RT) in patients with HNSCC.

## Results

### Development of prognostic gene expression signatures

HNSCC is the fourth cancer identified as having altered SOX2 expression (21%) (Supplementary Fig. 1A). SOX2 mRNA expression is significantly correlated with SOX2 copy number (correlation coefficient = 0.558, p = 3.54 × 10^−26^; Supplementary Fig. 1B), suggesting that altered SOX2 copy number is an important genetic event in HNSCC. SOX2 amplification is associated with several cancers^14–17^. Many samples lacking SOX2 amplification had high SOX2 mRNA expression (Supplementary Fig. 1B), suggesting that altered SOX2 copy number is not the only mechanism underlying SOX2 activation in HNSCC. Because many samples had high SOX2 expression in the absence of its amplification, we determined overlapping genes whose expression is correlated with SOX2 mRNA expression and copy number (Pearson correlation coefficient > 0.4 or < −0.4, and P < 0.001).

**Figure 1:**
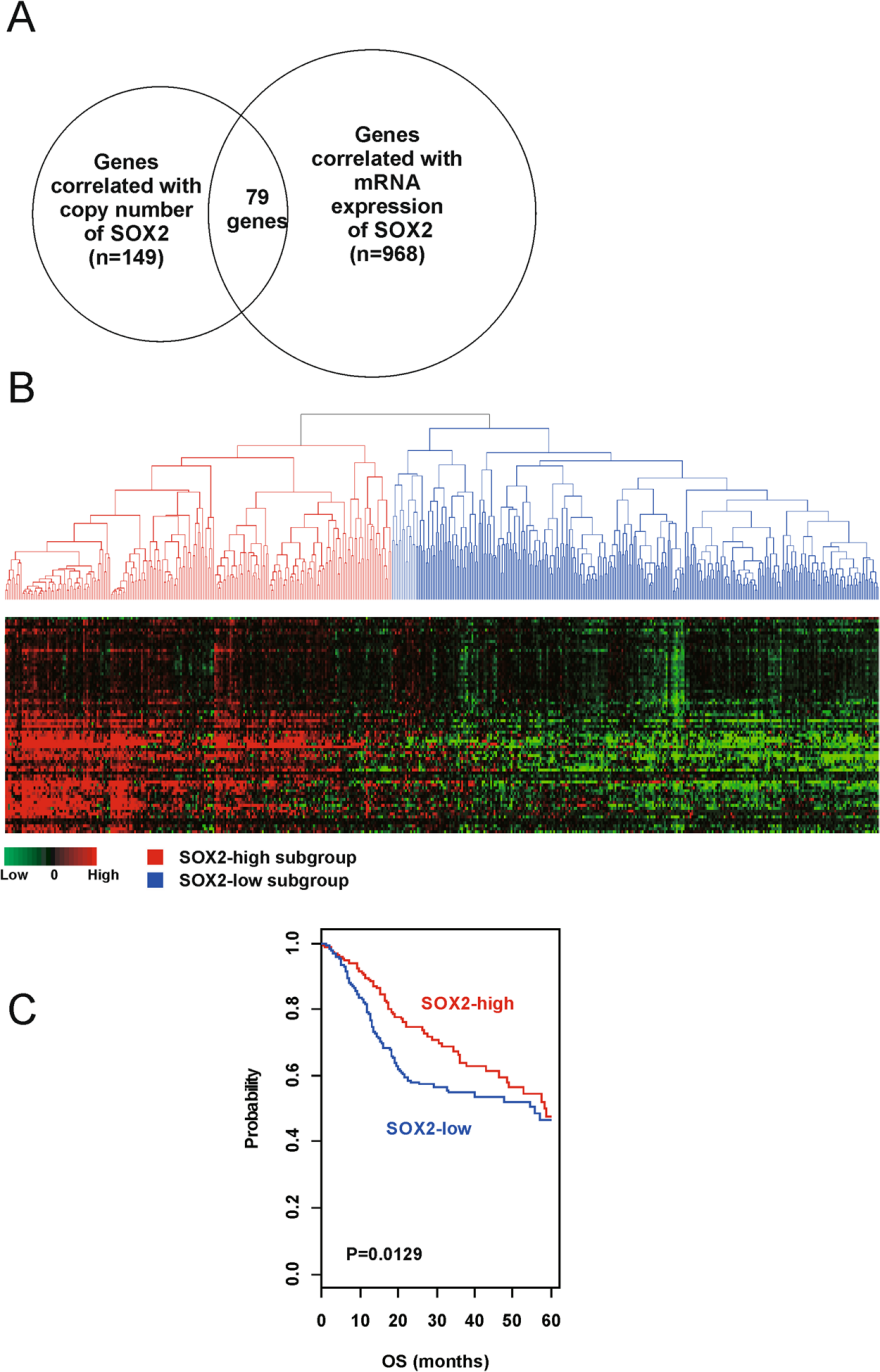
Stratification of HNSCC patients in the TCGA cohort according to SOX2 signature. (**A**) Venn diagram of genes selected by performing a double correlation analysis. (**B**) Hierarchical clustering of SOX2 expression data in the TCGA cohort. (**C**) Kaplan-Meier plots of overall survival (OS) of SOX2-high and SOX2-low subgroups in the TCGA cohort.

We identified 79 genes whose expression is significantly correlated with SOX2 copy number and mRNA expression that were part of the SOX2 signature in HNSCC and were used for constructing the prediction model (Fig. 1A and Supplementary Table 1). Patients were classified into two distinct subgroups (SOX2-high and SOX2-low subgroups) by performing hierarchical clustering analysis of gene expression data obtained from the training dataset (The Cancer Genome Atlas [TCGA] cohort, n = 513; Fig. 1B). Results of Kaplan-Meier analysis and log-rank test showed significant differences in the overall survival (OS) of patients in these two subgroups. The OS of patients in the SOX2-high subgroup was significantly longer than that of patients in the SOX2-low subgroup (p = 0.0129; Fig. 1C).

### Independent validation of the identified expression signature

**Figure 2:**
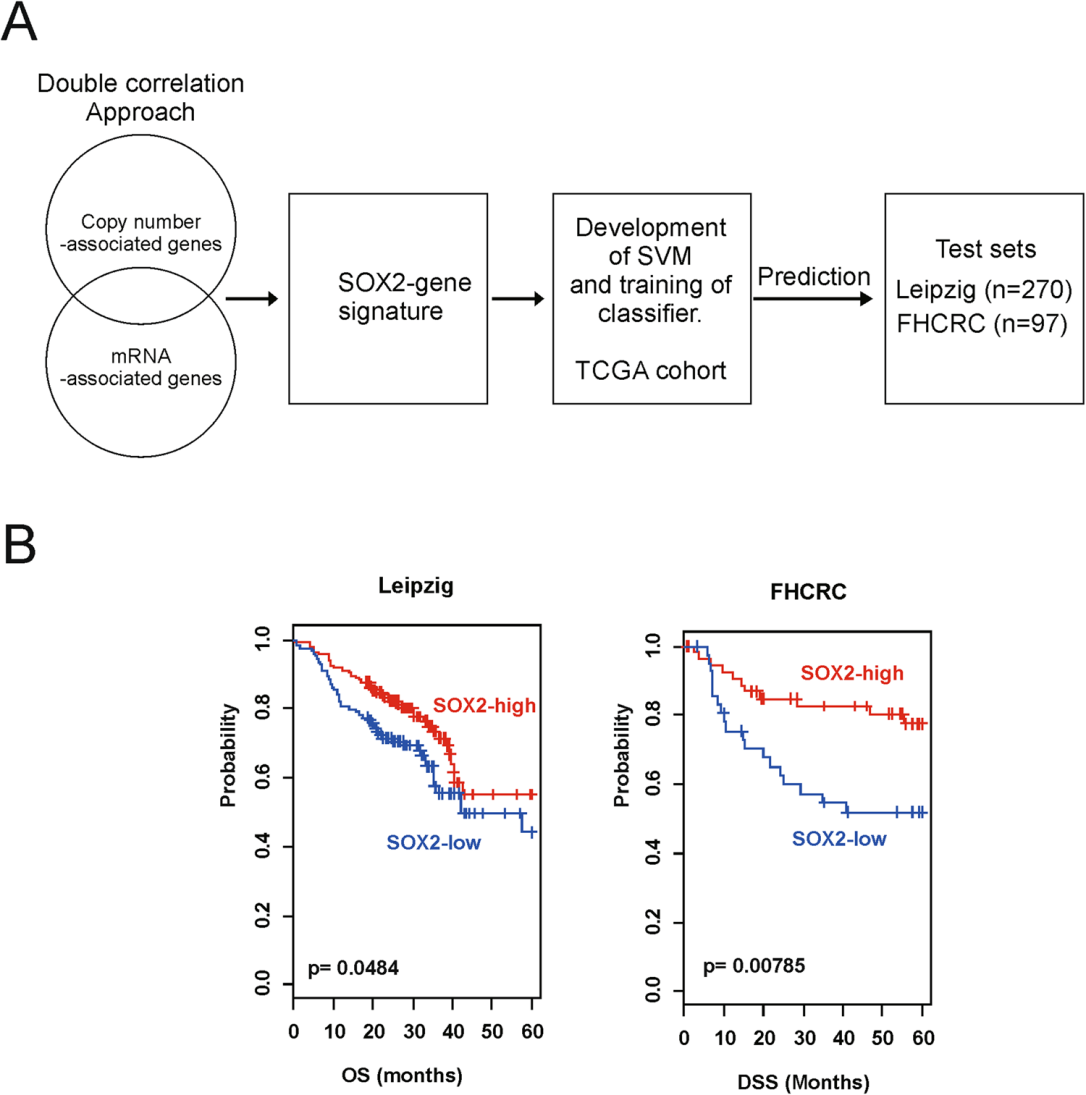
Construction of the prediction model and evaluation of predicted outcomes. (**A**) Schematic overview of the strategy used to construct prediction models and to evaluate predicted outcomes based on gene expression signatures. (**B**) Kaplan-Meier plots of the overall survival (OS) of patients in the Leipzig cohort. (**C**) Kaplan-Meier plots of disease-specific survival (DSS) of patients in the FHCRC cohort. Patients were stratified according to SOX2 signatures. Differences between the groups were significant, as determined by the log-rank test.

Robustness of the signature of the 79 genes was validated using two independent patient cohorts: the Leipzig cohort (n = 270) and the Fred Hutchinson Cancer Research Center (FHCRC) cohort (n = 97). A flow chart of the validation procedure is shown in Fig. 2A.

SOX2 signatures efficiently classified the patients into SOX2-high and SOX2-low subgroups according to the SVM classifier, which was consistent with the results obtained for the TCGA cohort. Classification of patients belonging to the Leipzig and FHCRC cohorts according to SOX2 signatures highlighted statistically significant differences in their prognosis in all independent validation data sets. Results of the Kaplan-Meier analysis and log-rank test showed significant differences in the OS of patients in the two subgroups in the Leipzig cohort (p = 0.0484; Fig. 2B) and in the DSS of patients in the two subgroups in the FHCRC cohort (p = 0.00785; Fig. 2B). These results support the prognostic value of SOX2 signature in the analysed cohorts.

### Association of SOX2 signature with clinicopathological variables

To evaluate the prognostic value of SOX2 signature and clinicopathological variables (e.g. patient age, sex, smoking status, alcohol consumption, anatomic site, primary tumour stage, lymph node (LN) metastasis, and TNM stage) of patients with HNSCC, we performed univariate and multivariate Cox proportional hazards regression analyses using clinical data of patients in the TCGA and Leipzig cohorts (n = 725). Results of the univariate analysis revealed that age (<60 years vs. ≥60 years), anatomic site (oropharynx vs. other sites), T stage (T1 and T2 vs. T3 and T4), and SOX2 signature (SOX2-high subgroup vs. SOX2-low subgroup) were significantly associated with OS. Results of the multivariate analysis showed that T stage and SOX2 signature were significantly independent prognostic factors (HR [95% CI], 2.07 [1.32–3.24]; p = 0.00156; HR [95% CI], 1.45 [1.09–1.92]; p = 0.01016; Table 1).

**Table 1:** Univariate and Multivariate Cox proportional hazard regression analysis of overall survival in the TCGA and Leipzig cohort (n = 725).

Variables	Univariate		Multivariate	
	HR (95% CI)	P-value	HR (95% CI)	P-value
SOX2 signature (SOX2^low^)	1.59(1.23–2.06)	0.00037	1.45(1.09–1.92)	0.01016
Gender (male)	0.81(0.61–1.08)	0.15	0.93(0.68–1.28)	0.66866
Age (>60 y)	1.31(1.02–1.69)	0.035	1.24(0.95–1.62)	0.12136
Smoking (YES)	1.03(0.76–1.40)	0.86	1.05(0.76–1.45)	0.77989
Alcohol (YES)	0.86(0.65–1.15)	0.32	0.99(0.72–1.36)	0.95990
Anatomic site (Oropharynx)	0.52(0.30–0.90)	0.020	0.62(0.36–1.08)	0.09303
Primary tumor (T3 & 4)	1.70(1.28–2.24)	0.000125	2.07(1.32–3.24)	0.00156
Regional lymph node (N +)	1.17(0.91–1.52)	0.21	1.29(0.93–1.79)	0.12835
Stage (stage III & IV)	1.32(0.95–1.82)	0.097	0.66(0.36–1.21)	0.17777

### SOX2 activation is correlated with the result of radiotherapy

**Figure 3:**
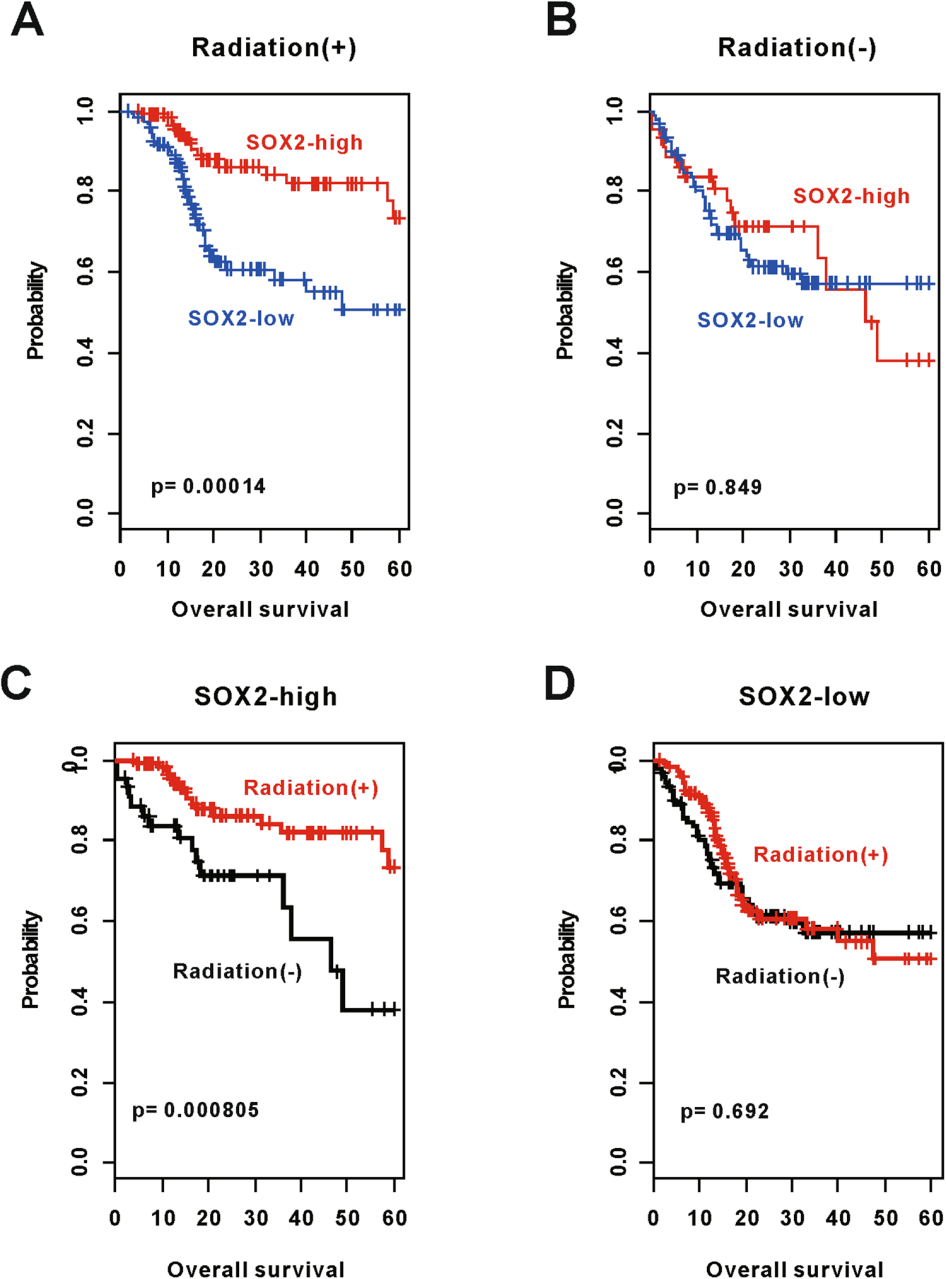
Association of SOX2 signature with radiotherapy (RT). (**A** and **B**) Subset analysis according to patients receiving RT. Patients were divided into subgroups based on SOX2 signatures. (**C** and **D**) Prediction of the response of patients in the two subgroups to RT according to SOX2 signatures. Patients in the SOX2-high subgroup benefited significantly from RT.

RT data were available for 384 of the 513 patients in the TCGA cohort. Of the 384 patients, 245 received radiation. The remaining patients did not receive RT (n = 139). Among the patients who received RT, patients in the SOX2-high subgroup had significantly longer OS than those in the SOX2-low subgroup (p = 0.00014; Fig. 3A). However, this difference in OS was not observed among patients who did not receive RT (p = 0.849; Fig. 3B). Response to RT was significantly high in patients in the SOX2-high subgroup (p = 0.000805; Fig. 3C). However, no significant benefit of RT was observed in patients in the SOX2-low subgroup (p = 0.692; Fig. 3D). Results of the univariate cox regression analysis showed that SOX2-related gene signature (p = 0.00048) and RT (p = 0.00978) were significantly associated with the survival of HNSCC patients. To determine any correlation between RT and SOX2-related gene signature, we performed an interaction test for OS. Results of the interaction test showed a significant correlation between SOX2 signature and RT (p = 0.018). We also performed multivariate Cox regression analyses to assess whether SOX2 signature has an independent prognostic value in HNSCC patients treated with RT. We found that SOX2 signature had only independent prognostic variables in patients treated with RT (HR [95% CI], 2.97 [1.45–6.09]; p = 0.00302).

### Association of SOX2 signature with other subtypes of HNSCC

To assess the association of SOX2 signature with clinically recognised subtypes of HNSCC, we performed subset analyses using the expression data of the TCGA and Leipzig cohorts (Supplementary Fig. 2). We assessed the association of SOX2 signature with tumour site, human papillomavirus (HPV) infection status, regional LN metastasis, T stage, and smoking status.

Comparing tumour sites revealed that 30.14%, 32.88%, 30.68%, and 6.30% of patients in the SOX2-high subgroup had oral cavity cancer, oropharynx cancer, larynx cancer, and hypopharynx cancer, respectively; and that 67.99% of patients in the SOX2-low subgroup had oral cavity cancer. Analysis of HPV infection status detected HPV-positive tumours in 98/313 (31.31%) patients and HPV-negative tumours in 215/313 (68.69%) patients in the SOX2-high subgroup. Furthermore, HPV-positive tumours were detected in 30/298 (10.07%) patients and HPV-negative tumours were detected in 268/298 (89.93%) patients in the SOX2-low subgroup. These results indicate that patients in the SOX2-high subgroup had a higher frequency of HPV infection than patients in the SOX2-low subgroup (p = 2.15 × 10^−10^). Next, we assessed the association between smoking and SOX2 signature and found that 57/364 (15.66%) patients in the SOX2-high subgroup were non-smokers and the remaining 307 patients (84.34%) were smokers. On the other hand, among the 396 SOX2-low subgroup patients, 105 (26.52%) were non-smokers and 291 (73.48%) were smokers (p = 3.68 × 10^−4^). However, no significant differences were observed in LN metastasis and T stage between patients in the SOX2-high and SOX2-low subgroups. We next assessed the association of SOX2 signatures with 4 previously reported molecular subtypes of HNSCC^18^. The SOX2-high subgroup corresponded with atypical (43.6%) and classic (38.2%) subtypes, and the SOX2-low subgroup corresponded with basal (58.6%) and mesenchymal (31.9%) subtypes (p = 2.2 × 10^−16^) (Supplementary Fig. 3). According to the molecular subtype of lung SCC, which is the basis of this classification, classic subtypes are known to be associated with xenobiotics metabolism, such as smoking. In the present study, the SOX2-high subgroup was associated with smoking, and corresponded with the classic subtype.

### Relationship between SOX2 signature and other genetic events

**Figure 4:**
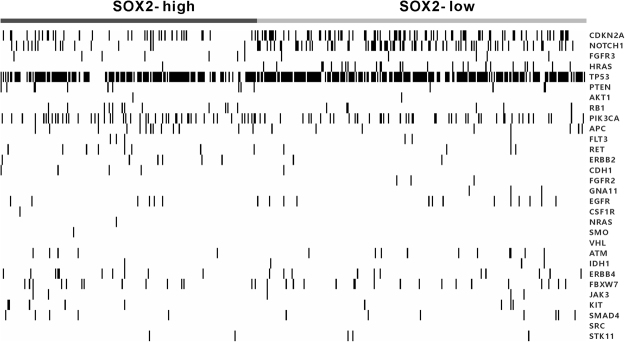
Somatic mutations in HNSCC cells according to SOX2 signatures of patients in the TCGA and Leipzig cohorts. Samples are shown in columns and are grouped according to SOX2 signature. P-values were obtained using the Fisher’s exact test.

To investigate the co-occurrence of somatic mutation and SOX2 activation in HNSCC, we analysed somatic mutation data of patients in the TCGA cohort (n = 493). We evaluated the frequency of somatic mutations in 30 genes associated with HNSCC. Among these genes, CDKN2A, FGFR3, HRAS, and TP53 had significantly different mutation rates between patients in the SOX2-high and SOX2-low subgroups (Fig. 4 and Supplementary Table 2). The frequency of somatic mutations was high in patients in the SOX2-low subgroup. However, no differences were observed in mutation rates of other genes between patients in the SOX2-high and SOX2-low subgroups.

### SOX2 overexpression enhances the effect of radiotherapy on HNSCC cell lines

**Figure 5:**
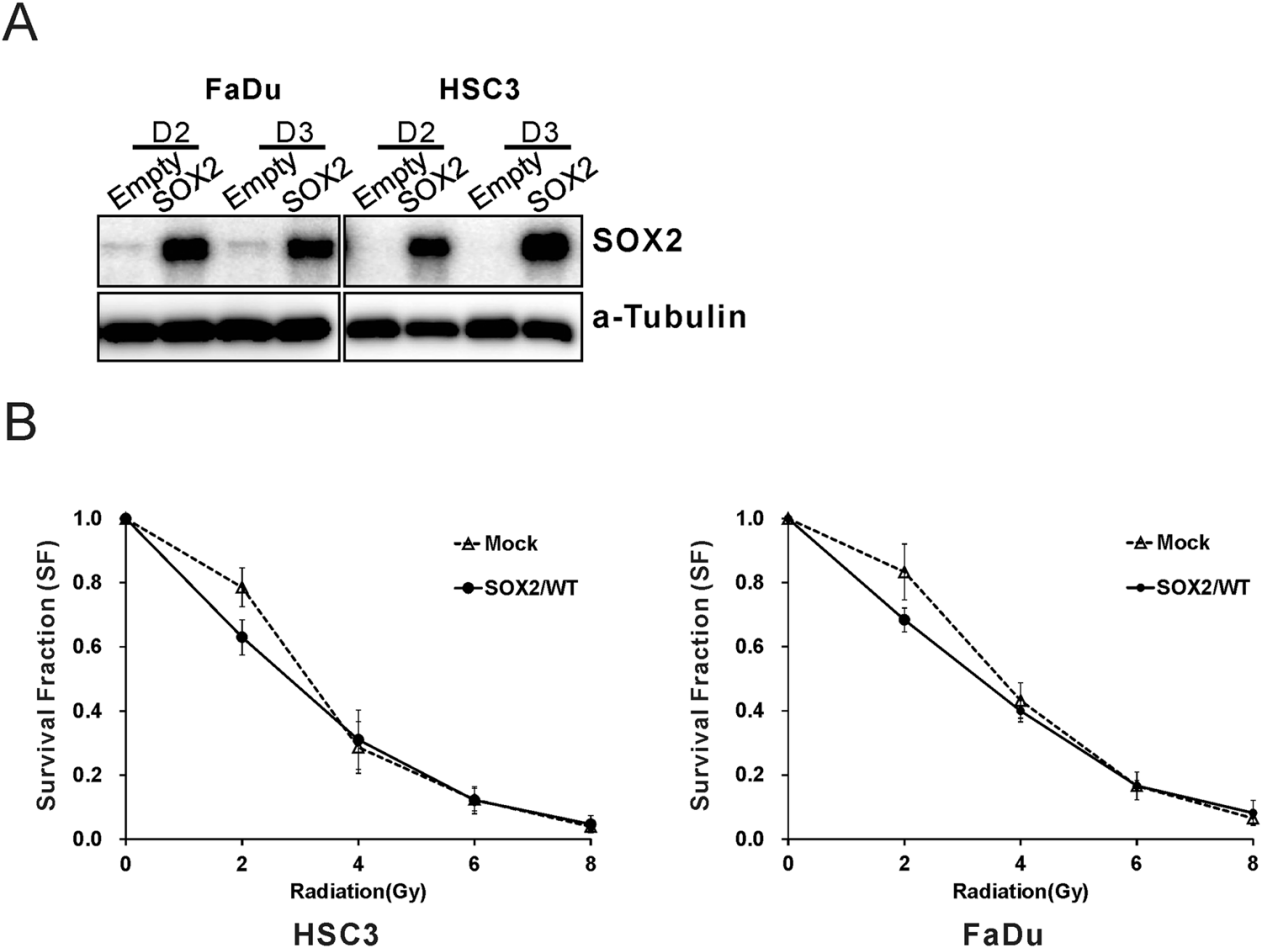
SOX2 overexpression enhances the effect of radiation on FaDu and HSC3 cells. (**A**) Western blot analysis. SOX2 expression levels were monitored in FaDu and HSC3 cells stably transfected with empty vector or vector expressing wild-type SOX2. (**B**) Survival rates of SOX2-overexpressing FaDu and HSC3 cells were significantly lower than those of empty vector-transfected FaDu and HSC3 cells after treatment with 2-Gy radiation.

To determine the role of SOX2 in response to RT, we generated stable FaDu and HSC3 cells overexpressing SOX2. SOX2 expression was higher in stably transfected cells than in mock-transfected cells (Fig. 5A). Next, we compared SF in stably transfected and mock-transfected cells irradiated with 2- to 8-Gy radiation by performing a clonogenic cell survival assay. Treatment with 2-Gy radiation significantly decreased the SF in SOX2-overexpressing cells compared with that in mock-transfected cells (FaDu cells, 0.63 vs. 0.83, p < 0.001; HSC3 cells, 0.63 vs. 0.79, p < 0.001; Fig. 5B). However, this effect was not observed after treatment with > 4-Gy radiation. Importantly, RT is typically performed using 2-Gy radiation. These results indicate that SOX2 overexpression enhances the effect of RT with 2-Gy radiation.

## Discussion

In the present study, we found that SOX2 signature is a predictor of HNSCC prognosis. We observed that patients in the SOX2-high subgroup had a better prognosis than those in the SOX2-low subgroup. Patients in the SOX2-low subgroup had HPV-negative tumours and had higher rates of somatic mutations in CDKN2A, FGFR3, HRAS, NOTCH1, and TP53 than patients in the SOX2-high subgroup. In addition, we observed that SOX2 signature could predict patient outcomes with RT, suggesting that SOX2 signature provides novel information for developing new biomarkers to assist in clinical decision making. This result was also confirmed using HNSCC cell lines.

SOX2 is a member of the SOX gene family that encodes transcription factors^4^ and is required for maintaining embryonic stem cells and for inducing cellular reprogramming^19^. A previous study identified OCT4, SOX2, and NANOG co-occupying a target gene set, and found that these 3 genes collaborated to form regulatory circuitry consisting of autoregulatory and feedforward loops^20^. Our SOX2 signature had 7 genes in common with the 353 genes identified by Bayer *et al*. We think that the difference is caused by the difference in the way the genes are selected and the methods used to find the genes. Studies performed to date have shown that ectopic expression and amplification of SOX2 are associated with the development of cancers, such as lung and breast cancers; however, the role of SOX2 in cancer is still controversial^5^. Some studies have suggested that SOX2 overexpression promotes cancer progression, suggesting that low SOX2 expression increases the survival rate of patients with cancer compared with high SOX2 expression. Schröck *et al*. suggested that SOX2 amplification is associated with poor prognosis of patients with HNSCC (including patients with advanced LN metastasis), and increases resistance to chemotherapy^12^. On the other hand, SOX2 amplification has been shown to be associated with favourable survival outcomes in many studies^21,22^. In addition, a recent meta-analysis showed that SOX2 expression is associated with a positive prognosis in non-small cell lung cancer^23^. However, some studies have reported that SOX2 suppresses tumour formation and that SOX2 overexpression inhibits cell proliferation^19^. Consistent with these results, Züllig *et al*. reported that low SOX2 expression is significantly associated with poor survival, and that high SOX2 expression is significantly associated with decreased LN metastasis and improved prognosis^13^. Bayo *et al*. reported that SOX2 inhibits tumour cell motility in HNSCC cells and that low SOX2 expression serves as a prognosticator to identify HNSCC patients at high risk for treatment failure^24^. High SEC. 62 and low SOX2 expression of chromosome 3q26-encoded genes were related to lymph node metastasis and worse prognosis in HNSCC and cervical cancer of unknown primary patients^25^. In this study, patients with high mRNA expression had a better prognosis than those with low mRNA expression (p = 0.0368) (Supplementary Fig. 4). These conflicting results may be associated with the heterogeneity of primary tumours and treatment modalities.

An association between SOX2 and a favourable prognosis has still not been demonstrated conclusively. Because normal squamous epithelia express SOX2, high SOX2 expression may reflect well-conserved differentiation, which is consistent with a report by Michifuri *et al*.^26^. Alternatively, SOX2 overexpression may occur early during HNSCC carcinogenesis and may be lost as the disease progresses because of genetic inactivation. Therefore, additional studies are required to elucidate the function of SOX2.

RT either alone or in combination with surgery or chemotherapy is an essential treatment modality for HNSCC. Because response to RT and clinical outcomes may differ between patients, factors associated with resistance to RT need to be identified. Previous studies have reported that SOX2 overexpression increases the radioresistance of cervical SCC, rectal cancer, and glioblastoma^27–29^. However, limited information is available on the relationship between SOX2 expression and the response of HNSCC to RT. Previous studies have shown that SOX2 overexpression increases the tumorigenic properties of oral SCC and that SOX2 downregulation decreased the tumorigenicity and radiochemoresistance of oral SCC both *in vitro* and *in vivo*^30^. Radiation-induced dedifferentiation of non-stem human papilloma virus (HPV)-negative HNSCC cells into cancer stem cells (CSCs) correlates with re-expression of reprogramming factors, such as OCT4 and SOX2^31^. It was found that the increase of CSCs was the cause of the poorer response to radiation treatment for HPV-negative HNSCCs than HPV-positive HNSCCs^31^. On the other hand, CSC frequency was found to be 62.5-fold greater in an HPV-positive tumour than in an HPV-negative tumour^32^. Another study reported that high nuclear SOX2 expression in early oral SCC was associated with prolonged disease-free survival after post-operative RT compared with low nuclear SOX2 expression^33^. We observed that patients in the SOX2-high subgroup had significantly improved outcomes after RT, whereas those in the SOX2-low subgroup did not significantly benefit from RT. Analysis using HNSCC cell lines showed that SOX2 overexpression enhanced the effect of RT. These conflicting results may be due to many factors that affect radiosensitivity, such as hypoxia, cell cycle, apoptosis, growth factors, CSCs, and genetic alterations in tumours^27^.

In the present study, patients in the SOX2-low subgroup had high rates of somatic mutations in CDKN2A, FGFR3, HRAS, NOTCH1, and TP53, which may be responsible for treatment-related heterogeneity. Ishigami *et al*.^34^ and Henson *et al*.^35^ reported that FGFR3 is associated with the radioresistance of oral SCC. FGFR3 expression levels were higher in radioresistant cells than in radiosensitive cells. FGFR3 may regulate radioresistance in human cancer cells by activating the Ras/mitogen-activated protein kinase pathway, which in turn upregulates the expression of several critical genes^36^. Several review studies have suggested that TP53 is important in the response to RT. Cells expressing wild-type TP53 are more sensitive to RT than cells expressing mutated TP53. TP53 mutations in HNSCC are associated with poor response to chemotherapy and RT, possibly because of the inhibition of radiation-induced senescence^37,38^. NOTCH1 functions as a tumour suppressor gene in HNSCC^39^ and is implicated in the radioresistance of glioma stem cells^40^. Activating mutations in one of the three RAS genes (HRAS, KRAS, and NRAS) are observed in 5–10% of patients with head and neck cancer, and HRAS mutations in particular are detected in > 50% of patients with head and neck cancer^41^. Although the precise mechanism is unclear, many studies have reported increased radioresistance in cells transfected with oncogenic RAS^42,43^.

Although mechanisms that regulate radiosensitivity in tumors are unclear, our study suggests that SOX2 signature can predict patients who might benefit from RT. Prediction of treatment outcomes helps in increasing the success of treatment and spares patients from receiving ineffective and harmful treatments. However, further studies are required to investigate the direct correlation between radioresistance and SOX2 activation.

In conclusion, our results suggest that SOX2 activation can help determine the prognostic risk in patients with HNSCC and can predict the response of these patients to RT.

## Methods

### Patients and gene expression data

This study included data from three independent patient cohorts. All clinical and gene expression data were collected previously and are available in public databases. Gene expression and somatic mutation data from TCGA (n = 513) were downloaded from the UCSC Cancer Genomics Browser (https://genome-cancer.ucsc.edu/). Data from the Institute for Medical Informatics, Statistics, and Epidemiology (Leipzig cohort [GSE65858], n = 270)^44^ and the FHCRC (GSE41613, n = 97)^45^ were obtained from the National Center for Biotechnology Information Gene Expression Omnibus database (http://www.ncbi.nlm.nih.gov/geo) and were used as test datasets. Gene expression data of the TCGA cohort were generated using Illumina HiSeq. 2000, those of the Leipzig cohort were generated using Illumina Human HT-12 V4.0 Expression BeadChip, and those of the FHCRC cohort were generated using Affymetrix Human Genome U133 Plus 2.0 Array. To compensate for the differences between each platform, all the data were standardised by being transformed into a median of 0 and standard deviation of 1. Pathological and clinical characteristics of patients in the three cohorts are summarised in Table 2.

**Table 2:** Clinical and pathological features of head and neck squamous cell carcinoma patients.

	TCGA cohort (N = 513)	Leipzig cohort (N = 270)	FHCRC cohort (N = 97)
Gender			
Male	370 (73.7%)	223 (82.6%)	66 (68.0%)
Female	132 (26.3%)	47 (17.4%)	31 (32.0%)
Age (mean ± SD)	60.9 ± 11.9	60.1 ± 10.0	NA
Anatomic site			
Oral cavity	301 (60.0%)	83 (30.7%)	86 (88.7%)
Oropharynx	79 (15.7%)	102 (37.8%)	11 (11.3%)
Larynx	113 (22.5%)	48 (17.8%)	0
Hypopharynx	9 (1.8%)	33 (12.2%)	0
others	0	4 (1.5%)	0
Primary tumor			
T1	33 (6.8%)	35 (13.0%)	NA
T2	147 (30.2%)	80 (29.6%)	NA
T3	129 (26.5%)	58 (21.5%)	NA
T4	178 (36.6%)	97 (35.9%)	NA
Regional lymph node			
N0	238 (49.5%)	94 (34.8%)	NA
N1	79 (16.4%)	32 (11.9%)	NA
N2	155 (32.2%)	132 (48.9%)	NA
N3	9 (1.9%)	12 (4.4%)	NA
Stage			
I	20 (4.1%)	18 (6.7%)	30 (30.9%)
II	96 (19.6%)	37 (13.7%)	11 (11.3%)
III	101 (20.7%)	37 (13.7%)	15 (15.5%)
IV	272 (55.6%)	178 (65.9%)	41 (42.3%)
HPV status			
Positive	68 (19.9%)	60 (23.4%)	0
Negative	274 (80.1%)	196 (76.6%)	97 (100%)
Tobacco use			
Never	114 (23.3%)	48 (17.8%)	NA
Yes	376 (76.7%)	222 (82.2%)	NA
Alcohol use			
Never	154 (42.1%)	31 (11.5%)	NA
Yes	212 (57.9%)	239 (88.5%)	NA
SOX2 signature			
SOX2-high	227 (44.2%)	146 (54.1%)	55 (56.7%)
SOX2-low	286 (55.8%)	124 (45.9%)	42 (43.3%)

Abbreviations: TCGA, The Cancer Genome Atlas; FHCRC, Fred Hutchinson Cancer Research Center; HPV, Human papilloma virus; NA, not available.

### Development of SOX2 signature

Gene expression data were analysed using BRB-Array Tools software program (http://brb.nci.nih.gov/BRB-ArrayTools/)^46^. Raw data were pre-processed using a robust multiarray averaging method for normalisation^47^.

SOX2-related genes in HNSCC were identified using a double correlation approach with the gene expression data from the TCGA cohort. SOX2-related genes were identified by determining Pearson correlation coefficients between the copy number of SOX2 and mRNA expression of each target gene, followed by the determination of the correlation between the mRNA expression of SOX2 and that of each target gene. Genes with P-values less than 0.001, and correlation coefficients greater than 0.4 or less than −0.4 were selected for analysis. We performed a hierarchical clustering analysis with the uncentered correlation coefficient as the measure of similarity and complete linkage clustering method. Patients were divided into two subgroups (SOX2-high and SOX2-low) based on the results of patient clustering analysis. Cluster analysis was performed using Cluster 3.0^48^.

### Construction of a prediction model and validation of the test datasets

To test the ability of gene expression signatures to predict the class of patients in another independent cohort, we used a previously developed model based on the support vector machine (SVM) algorithm^49^. Gene expression data of the training dataset (the TCGA cohort) were combined to obtain a series of classifiers according to the SVM algorithm. The robustness of the classifier was estimated based on the misclassification rate determined by performing leave-one-out cross-validation of the training dataset. Validation was performed using gene expression data obtained from two independent patient groups (the Leipzig and FHCRC cohorts).

### Statistical analysis

Gene expression data with available survival data were used to test the prognostic significance of SOX2 signatures. OS was defined as the time from surgery to death and was censored when a patient was alive at the last contact^50^. Disease-specific survival (DSS) was defined as the time between diagnosis and death due to disease-related causes or the last follow-up^51^. In the TCGA cohort, differences in response to RT were verified with an interaction test. Prognostic significance of SOX2 signature in patients in the SOX2-high and SOX2-low subgroups was estimated by performing Kaplan-Meier analysis^52^ and the log-rank test^53^. Univariate and multivariate Cox proportional hazards regression analyses were performed to evaluate independent prognostic factors associated with the survival of patients with HNSCC. Pearson correlation was used for correlation analysis, and Fisher’s exact test was used to assess the difference in the frequency of somatic mutations. P < 0.05 was considered statistically significant, and all statistical tests were two tailed. All statistical analyses were performed using R Project for Statistical Computing (http://www.r-project.org).

### Cell lines

HNSCC cell lines FaDu and HSC3 were purchased from Korean Cell Line Bank (Seoul, Korea). HSC3 cells were cultured in RPMI-1640 (Corning, Manassas, VA, USA), and FaDu cells were cultured in Eagle’s minimum essential medium (Corning, Manassas, VA, USA) supplemented with 10% foetal bovine serum (Corning, Manassas, VA, USA) and 1% penicillin-streptomycin (Corning, Manassas, VA, USA). All the cell lines were cultured at 37 °C under an atmosphere of 5% CO_2_.

### Plasmid transfection

At 24 hours before transfection, HNSCC cells were plated in a 60-mm dish and were cultured until they reached 60% confluency. Untagged plasmid empty vector (pCMV6_XL5) or SOX2 plasmid (pCMV6-XL5-SOX2, NM_003106.2) were purchased from Origene (Rockville, MD, USA). The cells were transfected with plasmids using TransIT-LT1 (Mirus Bio, Madison, WI, USA) following the manufacturer’s instructions.

### Western blot

HNSCC cells were irradiated, rinsed with ice-cold PBS, harvested using a cell scraper, and centrifuged. Cell pellets obtained were lysed by incubating on ice for 10 minutes, and cell lysates obtained were centrifuged at the maximum speed. Protein concentration was quantified with the Micro-BCA Protein Assay (Pierce, Meridian RD, USA). Next, equal amounts of protein mixed with loading dye were loaded in each lane of 10% or 12% SDS-polyacrylamide gel and were electrophoresed. The resolved proteins were transferred onto polyvinylidene difluoride membranes (Millipore, Billerica, MA, USA). The membranes were blocked and were probed using anti-SOX2 (Cell Signaling Technology, Beverly, MA, USA) or anti-α-tubulin (Abcam, Cambridge, MA, USA) primary antibodies, followed by incubation with horseradish peroxidase-conjugated secondary antibody (Cell Signaling Technology). Protein-antibody complexes were detected using an enhanced chemiluminescence kit (GE Healthcare. Buckinghamshire, UK) following the manufacturer’s protocol.

### Clonogenic survival assay

Exponentially growing cells were transfected with empty or SOX2-overexpressing plasmid before irradiation. Next, the cells were irradiated with 2-, 4-, 6-, or 8-Gy radiation using a Varian Clinac iX Linear Accelerator (Varian Medical Systems, Palo Alto, CA) at room temperature with 6 MV photon beam producing a dose rate of 6 Gray per minute. After 3 hours, the cells were trypsinised and were plated into 6-well cell culture plates containing a serum-containing medium. The cells were cultured for 10–14 days, fixed, and stained with Gentian violet. Colonies containing more than 50 cells were counted. To determine the cell survival percentage, colony-forming efficiency was determined, averaged, and normalised to that of non-irradiated control cells. Mean number of colonies obtained from three wells was corrected according to plating efficiency and was used to calculate cell survival at each dose of radiation. Cell survival curves for each radiation dose were fitted using a linear-quadratic model. An average (±SE) of normalised surviving fraction (SF) was determined from three independent experiments. Significance of the differences between dose responses was calculated using two-way ANOVA.

## Electronic supplementary material

Dataset1
