# Supplementary material for: SOX2 activation predicts prognosis in patients with head and neck squamous cell carcinoma
Source: Sci Rep. 2018 Jan 26;8:1677. doi: 10.1038/s41598-018-20086-w (PMC5785960; doi:10.1038/s41598-018-20086-w)
Supplement: Supplementary file 1 — Dataset1 [file 41598_2018_20086_MOESM1_ESM.pdf]

SOX2 activation predicts prognosis in patients with head and neck squamous cell carcinoma

Ji Hyun Chung[1], Hae Rim Jung[1], Ah Ra Jung[1], Young Chan Lee[1], Moonkyoo Kong[2], Ju-Seog Lee[3], Young-Gyu Eun[1,*]

[1]Department of Otolaryngology-Head and Neck Surgery, School of Medicine, Kyung Hee University

[2]Department of Radiation Oncology, School of Medicine, Kyung Hee University

[3]Department of Systems Biology, The University of Texas MD Anderson Cancer Center, Houston, Texas

Supplementary Figure Legends

Supplementary Fig. 1. SOX2 activation in HNSCC. (A) Cross-cancer summary of SOX2 alteration in 18 cancers using genome copy number data obtained from the TCGA cohort. HNSCC is the fourth cancer to have SOX2 amplification (21%). (B) Scatter plots of SOX2 mRNA expression and copy number alteration in patients in the TCGA cohort. SOX2 expression was substantially higher in samples lacking SOX2 amplification.

Supplementary Fig. 2. Association between SOX2 signature and other subtypes of HNSCC. Patients are shown in columns and are grouped according to SOX2 signature, tumour site, human papillomavirus (HPV) status, regional lymph node (LN) metastasis, T stage, and smoking status.

Supplementary Fig. 3. Frequency of molecular subtype between SOX2-high and SOX2-low subgroups.

Supplementary Fig. 4. Kaplan-Meier plots of overall survival (OS) of patients with HNSCC according to SOX2 mRNA expression in the TCGA cohort.

A

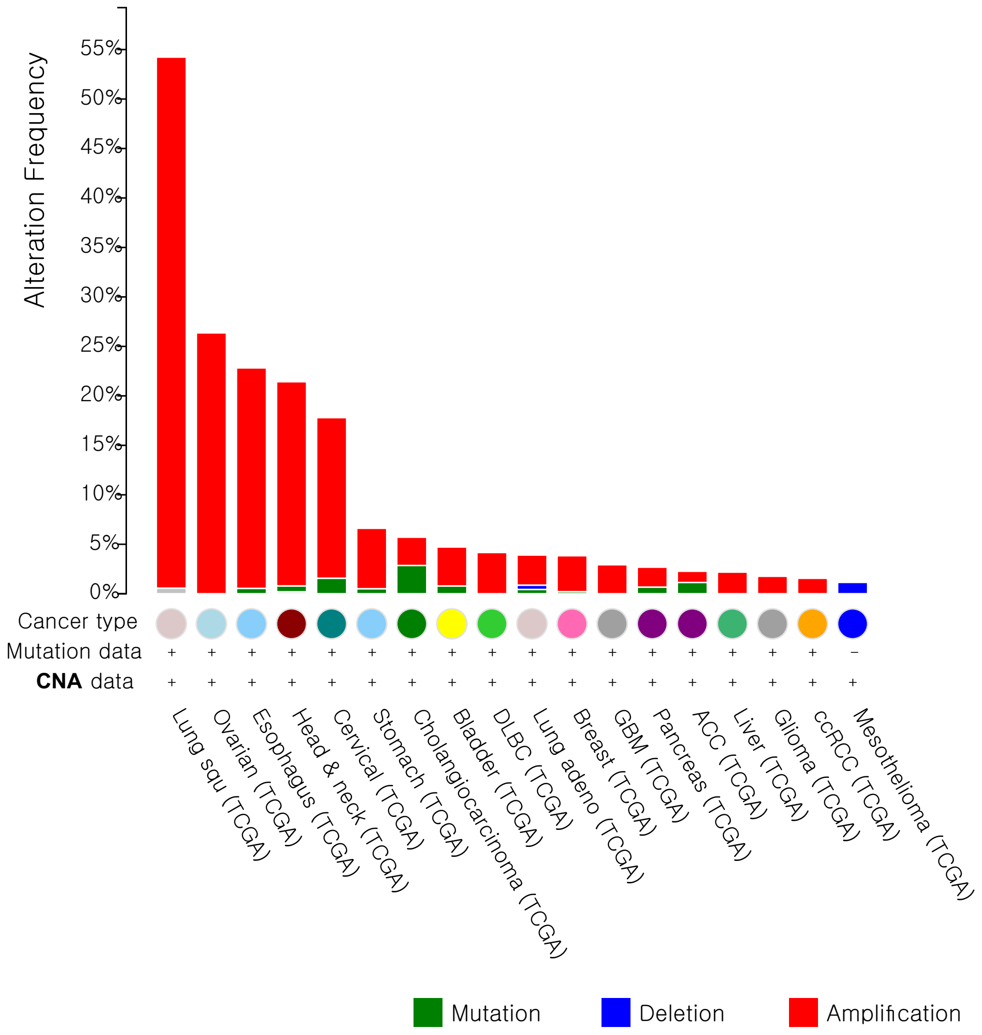

B

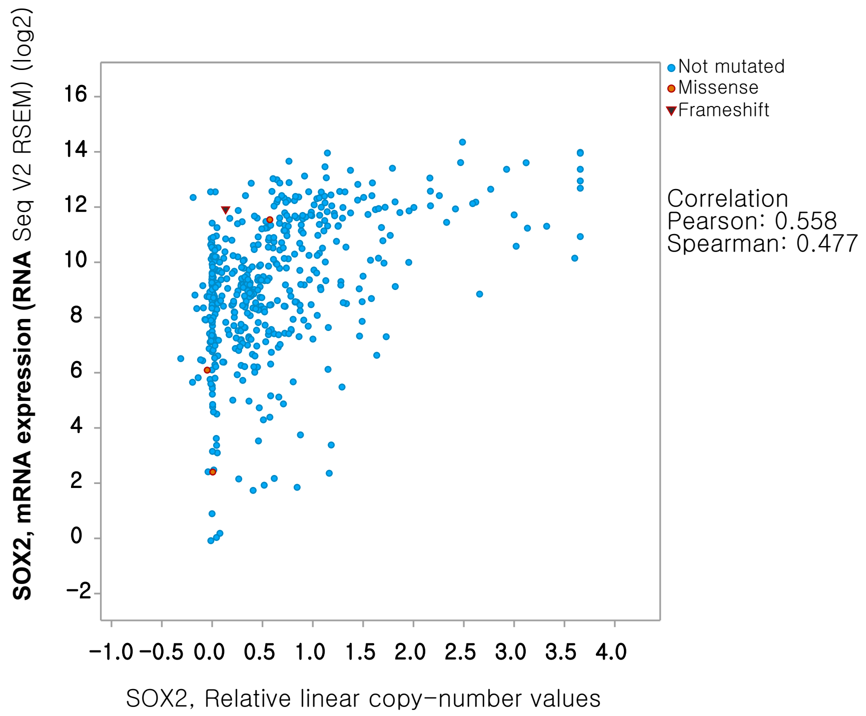

Suppl. Figure 1

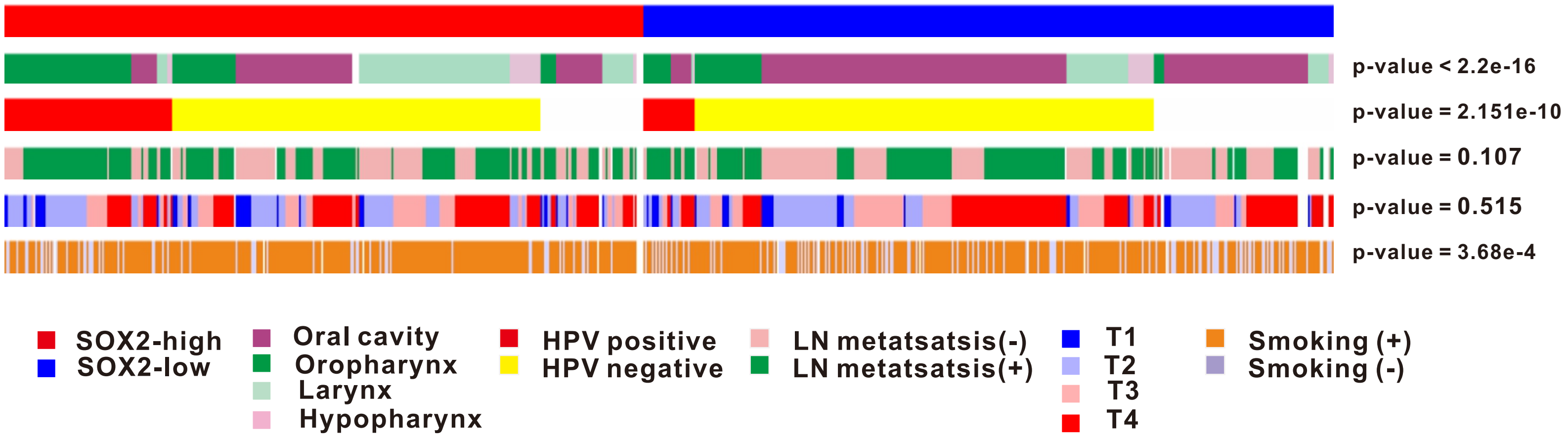

Suppl. Figure 2

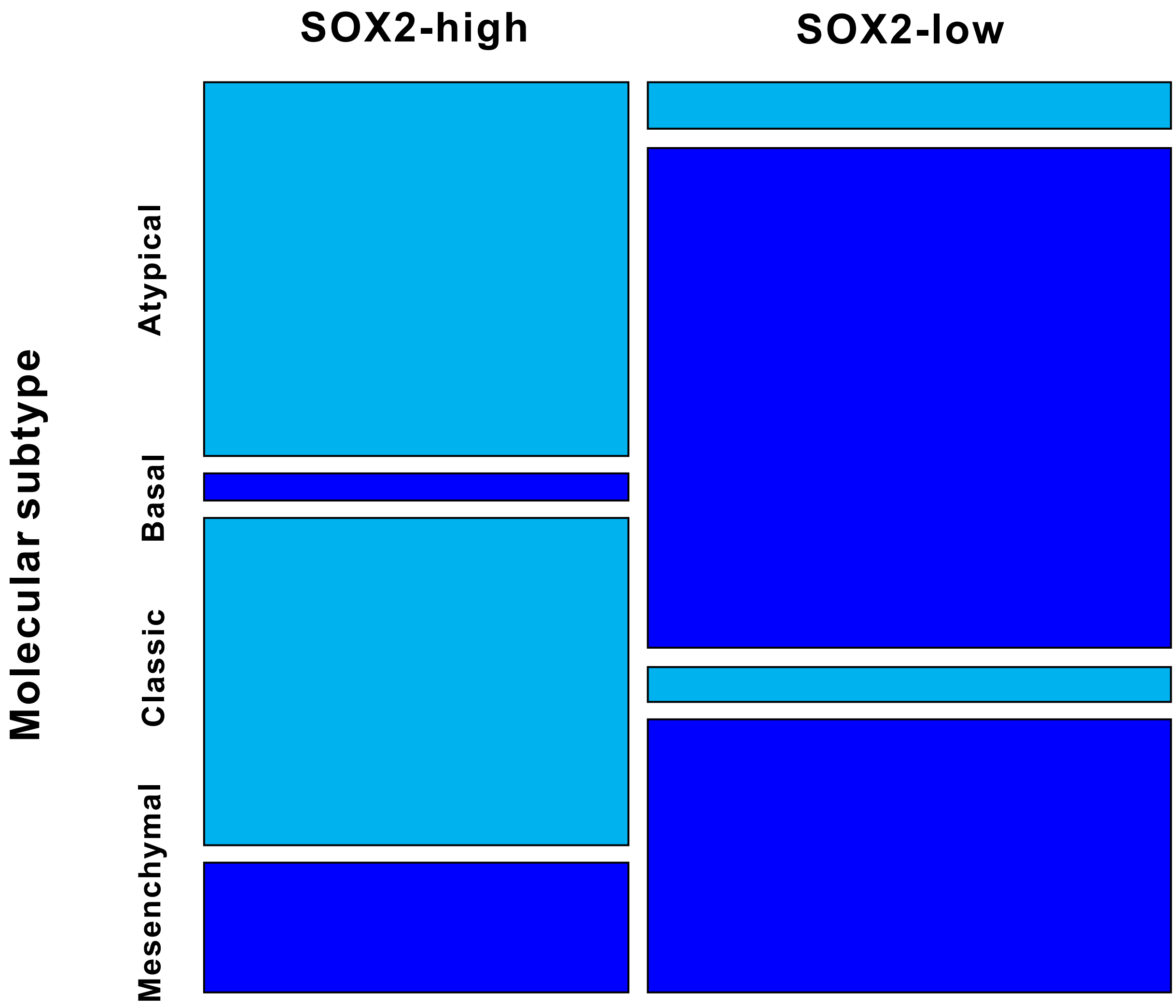

Suppl. Figure 3

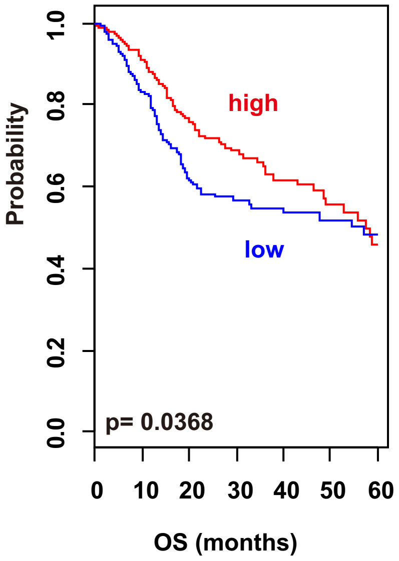

Suppl. Figure 4

Table S1. Genes associated with SOX2

TCGA RNA sequencing gene symbol	correlation coefficient with copy number of SOX2	p value of correlation with copy number of SOX2	correlation coefficient with gene expression of SOX2	p value of correlation with gene expression of SOX2
ABCC5	0.678	1.92523E-70	0.651	4.16604E-63
ACAP2	0.506	9.78107E-35	0.603	4.68376E-52
ACPL2	0.421	1.98927E-23	0.490	2.42246E-32
ACTL6A	0.738	1.74965E-89	0.471	1.00055E-29
ADRA2B	0.437	2.24913E-25	0.425	6.86321E-24
B4GALT4	0.437	2.39056E-25	0.529	2.56486E-38
BEX2	0.447	1.43944E-26	0.428	2.70066E-24
C3orf21	0.624	7.53622E-57	0.449	9.04895E-27
C3orf58	0.515	4.20488E-36	0.684	6.02487E-72
C6orf168	0.427	3.45506E-24	0.543	1.10478E-40
CACNA1B	0.446	2.13629E-26	0.506	8.68839E-35
CASP4	-0.413	1.63262E-22	-0.460	2.90495E-28
CEP70	0.449	8.2918E-27	0.521	5.32347E-37
CHST7	0.481	4.92114E-31	0.541	2.06789E-40
CLCN2	0.592	6.61921E-50	0.446	1.79782E-26
COCH	0.448	1.15867E-26	0.536	1.80041E-39
CYP26A1	0.404	1.28819E-21	0.601	1.07556E-51
DCUN1D1	0.635	2.30113E-59	0.406	8.46315E-22
DLG1	0.444	3.33888E-26	0.467	4.22562E-29
DLX6AS	0.405	1.0798E-21	0.511	1.50998E-35
DMRT3	0.405	1.02043E-21	0.505	1.23783E-34
DVL3	0.725	9.18162E-85	0.471	1.05205E-29

EIF4A2	0.499	1.0015E-33	0.423	1.02942E-23
EPCAM	0.415	8.05554E-23	0.537	1.12848E-39
FAM20B	0.434	5.36703E-25	0.427	4.14603E-24
FEZF1	0.426	5.3949E-24	0.492	1.39564E-32
GK5	0.412	2.02108E-22	0.446	1.81963E-26
GLT25D2	0.405	1.00949E-21	0.473	6.44051E-30
GPC3	0.404	1.41277E-21	0.472	6.70273E-30
GPR160	0.410	2.97186E-22	0.534	3.26647E-39
IFT80	0.503	3.13136E-34	0.422	1.32643E-23
IQCB1	0.418	4.21866E-23	0.506	1.11136E-34
KCNG3	0.428	2.91277E-24	0.514	6.0285E-36
KCNMB2	0.519	8.42383E-37	0.492	1.29582E-32
KCNMB3	0.631	2.39568E-58	0.441	7.58611E-26
KLHL24	0.524	1.39499E-37	0.455	1.54491E-27
LOC100130691	0.403	1.84918E-21	0.584	3.03542E-48
LSG1	0.676	6.02408E-70	0.465	5.82863E-29
MCCC1	0.686	1.03027E-72	0.607	5.70455E-53
MED12L	0.473	4.90775E-30	0.472	6.96423E-30
MRAP2	0.417	5.78204E-23	0.634	4.06282E-59
MSL2	0.485	1.12818E-31	0.503	2.92887E-34
MYNN	0.649	8.34691E-63	0.453	2.58657E-27
NCRNA00086	0.433	7.74741E-25	0.551	4.92812E-42
NMNAT3	0.410	2.95747E-22	0.520	5.88133E-37
NTRK2	0.415	8.83634E-23	0.764	1.2542E-99
NTS	0.424	7.56766E-24	0.619	1.0548E-55
OPA1	0.575	1.37104E-46	0.401	2.92893E-21
OR7A5	0.413	1.66114E-22	0.417	4.654E-23

PCCB	0.460	2.8295E-28	0.446	2.0147E-26
PHC3	0.552	2.56483E-42	0.442	5.69698E-26
PIR	0.405	1.20088E-21	0.593	5.22962E-50
PNCK	0.437	2.44554E-25	0.439	1.30532E-25
PRKCI	0.614	1.3106E-54	0.454	1.83606E-27
RAB6B	0.488	5.16015E-32	0.431	1.17188E-24
RASL11A	0.425	5.8441E-24	0.509	3.16502E-35
RIMKLA	0.405	1.22257E-21	0.557	3.96621E-43
RSRC1	0.501	5.75908E-34	0.467	4.26433E-29
SENP2	0.708	3.30702E-79	0.406	7.83144E-22
SGEF	0.439	1.42469E-25	0.712	1.4375E-80
SLC25A36	0.538	7.76435E-40	0.420	2.71419E-23
SOX2OT	0.607	4.3003E-53	0.797	6.2569E-114
STRBP	0.427	3.25372E-24	0.644	1.6076E-61
TBCCD1	0.644	1.99628E-61	0.458	5.55391E-28
TBL1XR1	0.726	3.81792E-85	0.467	3.42099E-29
TMEM20	0.415	8.64226E-23	0.493	9.52065E-33
TRA2B	0.502	3.50901E-34	0.443	4.48054E-26
TRIM59	0.480	6.78894E-31	0.423	9.85771E-24
TSEN15	0.443	4.26773E-26	0.449	8.30335E-27
ULBP1	0.440	9.56135E-26	0.401	2.7856E-21
VWA5B2	0.551	4.86855E-42	0.525	1.23116E-37
WNK2	0.414	1.03158E-22	0.603	3.61689E-52
ZNF639	0.787	1.4867E-109	0.419	3.40964E-23
ZXDC	0.420	2.18478E-23	0.419	3.09197E-23

Table S2. Comparison of somatic mutation frequencies between SOX2-high and SOX2-low subtypes.

		SOX-high	SOX-low	Fisher's p value
AKT1	MUT	1	1	0.054
	WT	215	276	
APC	MUT	13	8	0.115
	WT	203	269	
ATM	MUT	5	10	0.443
	WT	211	267	
CDH1	MUT	4	1	0.174
	WT	212	276	
CDKN2A	MUT	34	77	1.59E-07
	MUT	182	200	
CSF1R	WT	1	0	0.438
	MUT	215	277	
EGFR	WT	6	13	0.348
	MUT	210	264	
ERBB2	WT	6	2	0.146
	MUT	210	275	
ERBB4	MUT	10	11	0.823
	WT	206	266	
FBXW7	MUT	14	18	1
	WT	202	259	
FGFR2	MUT	0	3	0.26
	WT	216	274	
FGFR3	MUT	7	5	3.11E-06
	MUT	209	272	
FLT3	WT	3	3	0.115
	MUT	212	274	
GNA11	WT	0	3	0.26
	MUT	216	274	
HRAS	WT	1	26	3.11E-06
	MUT	215	251	
IDH1	MUT	1	3	0.635
	WT	215	274	
JAK3	MUT	2	3	1
	WT	214	274	

KIT	MUT	2	3	1
	WT	214	274	
NOTCH1	MUT	16	69	1.59E-07
	MUT	200	208	
NRAS	WT	1	0	0.438
	MUT	215	277	
PIK3CA	WT	47	43	0.079
	MUT	169	234	
PTEN	WT	10	4	0.053
	MUT	206	273	
RB1	MUT	12	6	0.054
	WT	204	271	
RET	MUT	8	4	0.142
	WT	208	273	
SMAD4	MUT	5	7	1
	WT	211	270	
SMO	MUT	1	0	0.438
	MUT	215	277	
SRC	WT	0	0	1
	MUT	216	277	
STK11	WT	2	3	1
	MUT	214	274	
TP53	WT	141	210	0.012
	MUT	75	67	
VHL	MUT	0	0	0.438
	WT	216	277	
